# Genetic Code Optimization for Cotranslational Protein Folding: Codon Directional Asymmetry Correlates with Antiparallel Betasheets, tRNA Synthetase Classes

**DOI:** 10.1016/j.csbj.2017.08.001

**Published:** 2017-08-12

**Authors:** Hervé Seligmann, Ganesh Warthi

**Affiliations:** a Aix-Marseille Univ, Unité de Recherche sur les Maladies Infectieuses et Tropicales Emergentes, UM 63, CNRS UMR7278, IRD 198, INSERM U1095, Institut Hospitalo-Universitaire Méditerranée-Infection, Marseille, Postal code 13385, France; b Dept. Ecol Evol Behav, Alexander Silberman Inst Life Sci, The Hebrew University of Jerusalem, IL-91904 Jerusalem, Israel

**Keywords:** Secondary structure, Codon-amino acid assignment, Mitochondrial genetic code, Synonymous codon, Alpha helix, Beta turn

## Abstract

A new codon property, codon directional asymmetry in nucleotide content (CDA), reveals a biologically meaningful genetic code dimension: palindromic codons (first and last nucleotides identical, codon structure XZX) are symmetric (CDA = 0), codons with structures ZXX/XXZ are 5′/3′ asymmetric (CDA = − 1/1; CDA = − 0.5/0.5 if Z and X are both purines or both pyrimidines, assigning negative/positive (−/+) signs is an arbitrary convention). Negative/positive CDAs associate with (a) Fujimoto's tetrahedral codon stereo-table; (b) tRNA synthetase class I/II (aminoacylate the 2′/3′ hydroxyl group of the tRNA's last ribose, respectively); and (c) high/low antiparallel (not parallel) betasheet conformation parameters. Preliminary results suggest CDA-whole organism associations (body temperature, developmental stability, lifespan). Presumably, CDA impacts spatial kinetics of codon-anticodon interactions, affecting cotranslational protein folding. Some synonymous codons have opposite CDA sign (alanine, leucine, serine, and valine), putatively explaining how synonymous mutations sometimes affect protein function. Correlations between CDA and tRNA synthetase classes are weaker than between CDA and antiparallel betasheet conformation parameters. This effect is stronger for mitochondrial genetic codes, and potentially drives mitochondrial codon-amino acid reassignments. CDA reveals information ruling nucleotide-protein relations embedded in reversed (not reverse-complement) sequences (5′-ZXX-3′/5′-XXZ-3′).

## Highlights

• Codon directional asymmetry, CDA, is zero for palindromes (XZX), -/+ 1 for ZXX, XXZ.
• CDA fits a tetrahedral genetic code representation.
• tRNA synthetase classes I/II associate with CDA direction.
• Antiparallel betasheet conformational indices of amino acids correlate negatively with mean CDA.

## Graphical Abstract

Fujimoto's tetrahedral genetic code representation and codon directional asymmetry, CDA.

**Figure:**
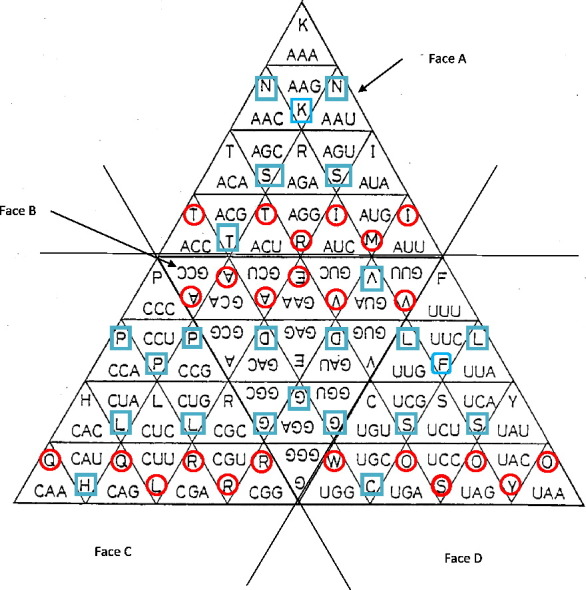

## 1 Introduction

The genetic code is optimised along several dimensions. Correlations between codon and amino acid properties have frequently been interpreted as resulting from evolutionary optimizations of the genetic code's codon-amino acid assignments. These minimise effects of: replicational/transcriptional nucleotide substitutions on amino acid hydrophobicity [1], [2], [3], [4], [5], [6], [7], [8], [9], [10], [11] and along multiple properties [12]. The genetic code is also optimised in relation to other processes, such as tRNA misloading with non-cognate amino acids [13], [14], [15], [16]; ribosomal frameshifts [17], [18], [19], [20], [21], [22], [23]; and protein folding kinetics [24], [25], [26].

Another approach assumes that the genetic code coevolved with codon/amino acid metabolic pathways [27], [28], [29], [30], [31]. It remains unclear whether genetic code optimizations are circumstantial byproducts of the metabolic coevolution hypothesis [32], [33], [34], [35], [36], or whether some combination of both processes produced the genetic code [34], [37], [38], [39], [40], [41], [42].

Here we present a previously unknown dimension of the genetic code. Analyses suggest that the genetic code is optimised in relation to this new property. The property reflects differences between nucleotides at first versus second codon positions, as compared to differences between nucleotides at third versus second codon positions. In this context, previous analyses [43] showed that the subtraction of dipole moments of nucleotides at first and second codon positions correlate with hydrophobicities of corresponding amino acids, after accounting for another, previously reported, correlation between codon and amino acid hydrophobicities [44], [45]. Here analyses generalise the principle to all codon positions and nucleotide properties.

## 2 Codon Directional Asymmetry

The new codon property is derived from comparing two differences in nucleotide contents, the difference between nucleotides at first and second codon positions, and the difference between nucleotides at second and third codon positions. This defines a codon's directional asymmetry in nucleotide content, CDA. CDA reflects semi-quantitatively extents by which a nucleotide at either 5′ or 3′ codon extremity differs from the codon's two remaining nucleotides. Along this principle, palindromic codons with the same nucleotide at 5′ and 3′ extremities (at first and third positions, XZX (including codons with X = Z)) are symmetric, CDA = 0. When the nucleotide at the 5′ extremity belongs to a different nucleotide group (purine/pyrimidine) than the two other positions and the latter are identical (ZXX), CDA = − 1. When the nucleotide at the 3′ extremity differs from other positions (XXZ), CDA = + 1. Signs for 5′-and 3′-dominant CDAs are arbitrary, but necessarily opposite (positive versus negative).

### 2.1 Purines and Pyrimidines

For codons of types ZXX/XXZ, CDA = − 0.5/+0.5, when both X and Z are purines, or both pyrimidines. This reflects lesser purine-purine and pyrimidine-pyrimidine structural differences than for purine-pyrimidine comparisons. This principle assigns a CDA score also for some codons of type XZW, where all three nucleotides differ, and Z belongs to the same chemical group (purine or pyrimidine) as the nucleotide at either codon extremity. For codons where nucleotides Z and W are both purines/pyrimidines, X is the most different nucleotide (CDA = − 0.5), because chemical structural differences between X and Z are greater than between W and Z. According to that rationale, for codons where nucleotides X and Z are both purines (or both pyrimidines), W is the most different nucleotide (CDA = + 0.5).

### 2.2 Complementarity Between Nucleotides at Different Codon Positions

For some codons with structure XZW, Z does not belong to the same group (in terms of purines/pyrimidines) as any nucleotide at the other positions. In these cases, an additional rule determines which of the nucleotides among X or W, differs more from the two others. We propose that complementarity between canonical base pairs (C:G and A:T/U) defines that complementary nucleotide pairs are the most different pairs. Hence for codons with structure XZW, CDA = − 0.5 and CDA = + 0.5 when X is the canonical complement of Z, and when W is the complementary of Z, respectively. This rule set defines CDA for all 64 codons (Table 1).

**Table 1:**
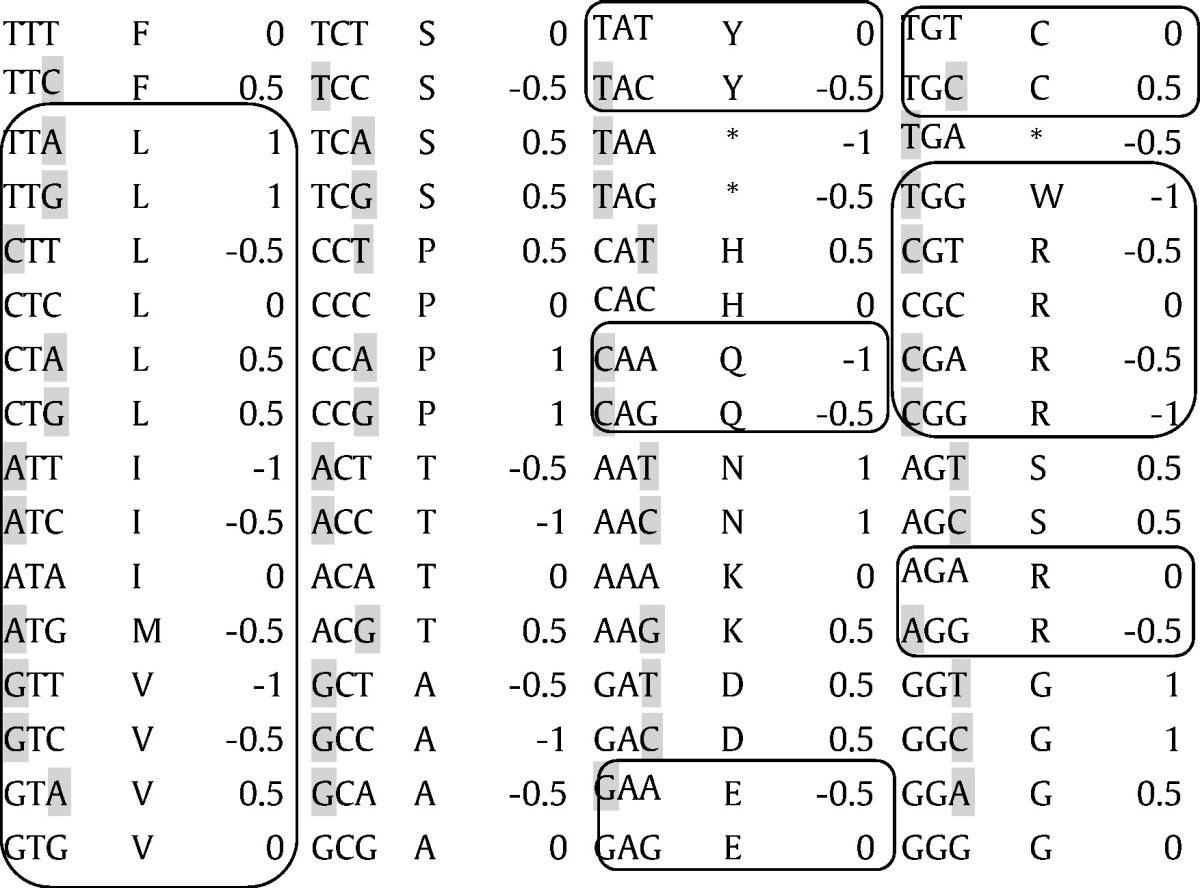
The genetic code's 64 codons and their codon directional asymmetry, CDA. Shaded nucleotides indicate the nucleotide at one of the codon's extremities that is the most different from nucleotides at other positions, along rules described in text, and which determines the dominant side of codon directional asymmetry: negative CDA when the first (5′) codon position has the most different nucleotide, and positive CDA when the third (3′) position has the most different nucleotide. Codons assigned to amino acids aminoacylated by class I tRNA synthetases are framed, remaining amino acids are aminoacylated by class II tRNA synthetases.

## 3 A New Dimension of the Genetic Code

The distribution of CDA in Table 1 is symmetric. Therefore, the genetic code table could probably be reordered so as to reveal graphically this symmetry, as done for other symmetry properties of the genetic code [46].

To what extent does CDA represent a dimension of the genetic code that is independent of other dimensions? In this respect, we compare Table 1 with the binary representation of the genetic code [47, therein figure 6], a rather complete 6-bit representation of each codon. It assigns to each codon position two binary values, the first representing the purine-pyrimidine divide, the second value represents whether the nucleotide forms two or three hydrogen interactions when in duplex conformation with an inverse-complementary strand. This defines two binary variables for each codon position, hence six binary variables for each codon.

Pearson correlation coefficients r of CDA with any of these six binary codon properties are ‘zero’, indicating that CDA is independent of each of these properties. Correlations with sums and subtractions between any pairs of these six binary values also yield r = 0. Results are identical if one pairs nucleotides according to keto versus amino nucleotides as previously reported [47], [48]. This means that CDA catches a genetic code dimension that differs from classically recognised codon properties.

### 3.1 Tetrahedral Representations and CDA

The genetic code can also be presented as a tetrahedron, with four equal triangular faces each subdivided into 16 equilateral, smaller triangles, representing the 64 codons. Castro-Chavez [49] reviews these representations, and proposes a tetrahedral representation, placing codons so that hydrophobic amino acids are central to each tetrahedral face, named faces A–D. Applying CDA to Castro-Chavez's tetrahedral representation, faces A and D tend to have CDA < 0, and faces C and B CDA > 0. Within each face, in total 19 triangle vertices (over all 4 faces) with CDA < 0 are common with vertices belonging to triangles with CDA > 0. This is very close to the 18 vertices expected if codons were randomly distributed in relation to CDA (*P* > 0.5, chi-square test), considering that 24 codons have CDA < 0, 16 have CDA = 0, and 24 have CDA > 0. Eleven among 24 vertices common between triangles from different faces of the tetrahedron are for triangles/codons with opposite CDA. This is slightly more than the 6.75 expected by random CDA distribution (*P* = 0.054, chi-square test). Hence the tetrahedral representation of Castro-Chavez [49] is random in relation to CDA within tetrahedral faces, and probably also between faces.

**Fig. 1:**
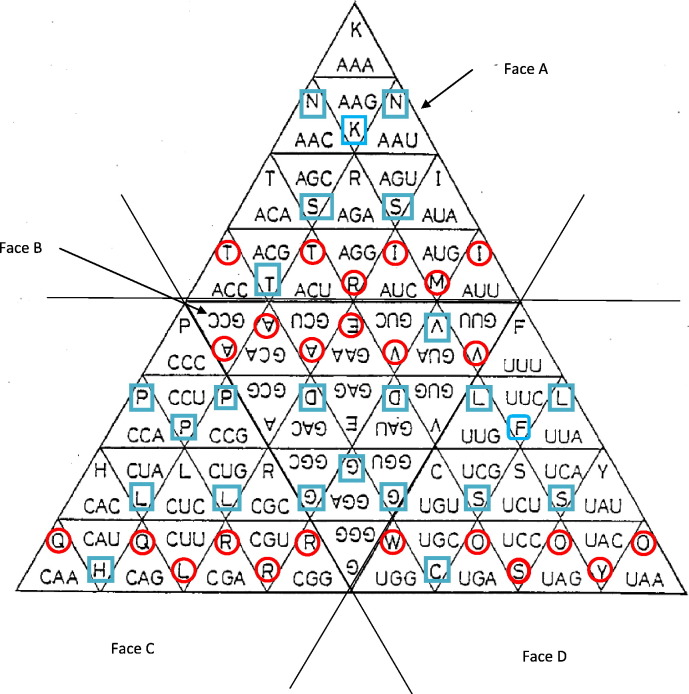
Fujimoto's tetrahedral codon stereo-table, a genetic code's representation that seems non-random in relation to codon directional asymmetry. The tetrahedron has four equal, equilateral faces (A–D), and consist each of 16 equilateral triangles representing each one codon. Red circles: CDA < 0; blue squares: CDA > 0. (For interpretation of the references to colour in this figure legend, the reader is referred to the web version of this article.)

Adapted from Fig. 1 at http://www.google.com/patents/US4702704.

Fujimoto's tetrahedral codon stereo-table [50] is much more ordered in relation to CDA's distribution among and within tetrahedral faces (Fig. 1): Faces A–D each have six codons with CDA < 0, six codons with CDA > 0, and four codons with CDA = 0. Within each face, there are exactly two contacts between codons/triangles with opposite CDA. This total of eight contacts between triangles with opposite CDA is significantly less than the expected 18 contacts for randomly distributed CDA within faces of the tetrahedron (*P* = 0.018, chi-square test). There are no contacts between tetrahedron faces for codons/triangles with opposite CDA (*P* = 0.0096, chi-square test). Hence Fujimoto's tetrahedral representation is most compatible with the genetic code's symmetries implied by CDA in Table 1.

The specific examples used here illustrate randomness versus CDA, and close to perfect reorganisation of the genetic code in relation to CDA, respectively. Other representations might reorganise the genetic code more optimally in relation to CDA. However, these representations may not relate to interpretable phenomena in the real world.

### 3.2 Codon Directional Asymmetry and Codon Participation in Error Correcting Codes

Genetic codes include a subjacent punctuation code called the natural circular code that enables retrieving the ribosomal translation frame [51], [52], [53], [54], [55]. Mechanisms for coding frame retrieval remain unknown, but are probably associated with circular code motifs conserved in tRNAs and ribosomal RNAs [56], [57], [58], [59]. Codon symmetry is particularly informative in relation to frame retrieval, as codons of type XZX (CDA = 0) have maximal capacity for reading frame retrieval [55], [60], [61], and have highest occurrences within various types of error-correcting codes [62]. Absolute values of CDA are lower for codons belonging to the natural circular code than for the remaining codons (*P* = 0.016, two tailed Mann-Whitney test). This principle is confirmed also when comparisons imply only codons belonging to the natural circular code: their absolute CDA increases with codon-specific reading frame retrieval (r = − 0.615, *P* = 0.002; rs = 0.44, *P* = 0.026, one tailed tests). Hence processes determining the near-universal natural circular code probably contributed biological functions to CDA.

## 4 Codon Directional Asymmetry and tRNA Synthetase Classes

CDA in Table 1 reflects a genetic code symmetry that does not follow the purine-pyrimidine, keto-amino, nor the weak-strong base-pairing patterns. A little known symmetry within the genetic code relates to Rumer's transformation [63], [64], [65], which replaces systematically all adenine (A) with cytosine (C) and vice versa, and also all guanine (G) with thymine (T) and vice versa. It is one among 23 bijective transformations [60], also called systematic nucleotide exchanges [66], [67] or ‘swinger’ transformations [68], [69], [70], [71]. RNA and DNA sequenced by several different methods and published in GenBank by various groups match these transformations. Hence while *a priori*, transformations such as Rumer's seem theoretical processes, they reflect biological realities, such as actual nucleotide sequences that were presumably produced by replication or transcription that systematically inserts a specific nucleotide instead of another specific nucleotide. This phenomenon of systematic nucleotide exchanges has similarities with isolated nucleotide misinsertions [60], [66], [67].

Rumer's transformation also correlates with a notable biological property, tRNA synthetase classes [72] of amino acids assigned to codons. The tRNA synthetases are enzymes that load amino acids to their cognate tRNA. The twenty tRNA synthetases form two groups of equal size, tRNA synthetase classes I and II based on structural homology [73], [74]. tRNA synthetases class I covalently link cognates to the 2′ hydroxyl group of the tRNA's last ribose, and class II to its 3′ hydroxyl group [75], [76].

The symmetry in the genetic code that correlates with tRNA synthetase classes exchanges nucleotides at the first and third codon positions along rule A ↔ C + G ↔ T (Rumer's transformation), and A ↔ G + C ↔ T at the second codon position. If instead of applying the nucleotide exchange rule A ↔ C + G ↔ T to the third codon position, one applies the exchange rule A ↔ T + C ↔ G, the symmetry between codons whose corresponding tRNA is aminoacylated by tRNA synthetase class I or class II is also recovered [77]. These symmetries by nucleotide exchanges are not mere theoretical considerations. Homologies of some DNA and RNA sequences in GenBank were detected after accounting for systematic nucleotide exchanges for the mitogenome [66], [67], [68], [69], [70], [71], [78], [79], [80]. In addition, the regular human mitogenome includes numerous repeats that can only be detected when assuming systematic exchanges [81], including palindromes [82].

CDA associates with tRNA synthetase classes. On average, codons assigned to amino acids aminoacylated by tRNA synthetases class I have CDA < 0 (15 among 21 codons (stops excluded), *P* = 0.039, two tailed sign test). For tRNA synthetases class II, the situation is opposite: most codons have CDA > 0 (17 among 24, CDA = 0, *P* = 0.032, two-tailed sign test). Sign tests are inadequate to handle codons with CDA = 0, therefore codons with CDA = 0 are excluded from these calculations. Mean CDA for tRNA synthetase classes differ significantly (two-tailed *P* = 0.002 for each *t*-test and Mann-Whitney test). These comparisons between means include codons with CDA = 0.

CDAs are averaged for codons assigned to specific amino acids. Mean CDA < 0 for 8 among 10 amino acids for class I; and CDA > 0 for 8 among 10 amino acids for class II (*P* = 0.006, two-tailed sign test for each tRNA synthetase class). Exceptions are Cys and Leu for class I, and Ala, and Thr for class II. Overall, the sign of mean CDA for codons assigned to an amino acid follows expected patterns (class I, CDA < 0; class II, CDA > 0) for 16 among 20 amino acids/tRNA synthetases (*P* = 0.00296, one tailed sign test).

Note that stop codons have CDA < 0, predicting tRNA synthetase class I. However, the tRNA synthetase of pyrrolysine, which is inserted at some stop codons, belongs to tRNA synthetase class II [83]. Exceptions might reflect historical constraints on the genetic code's genesis [77].

Hence the rationale defining CDA reveals a symmetry that is close to that of the combination of nucleotide exchanges that reveal the genetic code's symmetry in relation to tRNA synthetase classes. However, the rationale behind CDA is simpler and perhaps more amenable to mechanistic reduction.

### 4.1 Alternative Scores for Codons with CDA = | 0.5 |

Three different types of codons get CDA = | 0.5 |, based on different rationales: (a) codons with structures ZXX/XXZ where both X and Z are purines/pyrimidines; (b) codons with structure XZW where Z belongs to the same nucleotide family (purine/pyrimidine) as either X or W; and (c) codons with structure XZW where Z belongs to a different nucleotide family than X and Z. This scoring is somewhat arbitrary, and might not be optimal to reflect biological properties. Keeping signs, we rescore each of these three codon types with values | 0.25 | and | 0.75 |, resulting in different scoring systems for these three codon groups: alternative CDAs of groups (a, b, c) are (0.5, 0.25, 0.75), (0.5, 0.75, 0.25), (0.25, 0.5, 0.75), (0.25, 0.75, 0.5), (0.75, 0.5, 0.25), and (0.75, 0.25, 0.5). CDA of codons with CDA = 0 and CDA = | 1 | remain unchanged. These different scoring systems do not alter the strength of the CDA-tRNA synthetase class association: according to all these scoring systems, the same 8 among 10 codon families in class I have CDA < 0, and 8 among 10 amino acids in class II have CDA > 0. Excluding palindromic codons (CDA = 0) from calculations does not change results.

This heuristic approach suggests that associations between tRNA synthetase classes (an ancient property of the translational apparatus) and CDA are robust in relation to CDA's semi-quantitative scoring.

## 5 Translation Kinetics

The tRNA synthetase classes differ in the position of aminoacylation of the amino acid on the tRNA's acceptor stem. This probably affects the spatial kinetics of peptide elongation. We suggest that CDA also affects the spatial kinetics of codon-anticodon interactions in the ribosome's translational core (site P [84]; site A [85]). Hence both tRNA synthetase class and CDA would affect cotranslational protein folding, meaning folding during the process of peptide extension by ribosomal translation [86], [87], [88], [89], [90], [91], [92], [93], [94], [95], [96], [97]. Tentatively, we consider that associations between CDA and tRNA synthetase classes suggest synergistic effects on cotranslational protein folding by each CDA and tRNA synthetase class.

Note that cotranslational protein folding does not occur for all proteins [98]. Cotranslational protein folding frequently increases the yield of proper folds, but is not always an absolute requirement [99], [100], [101], [102], [103]; yet decreases misfolding probabilities [104], [105], [106]. Among others, at least in some cases, cotranslational folding requires complete protein structural subdomains [107], [108]. Cotranslational protein folding following the sense of translation (from the N terminal) predicts more accurately protein structures than when proceeding in the opposite sense (from the C terminal) [109], [110], indicating that cotranslational protein folding is a reality for most proteins. Nevertheless, cell free protein folding shows that cotranslational folding is not always required [111].

mRNA properties affecting translation speed and ribosomal pausing [112], [113], [114], also affect protein folding independently of that protein's amino acid sequence. Synonymous codons associate with different types of protein secondary structures [115], [116], in particular for clusters of rare codons on mRNAs [117], [118], [119]. These associations might explain effects of synonymous single nucleotide polymorphisms on protein function [120], [121], [122], [123] and are in line with selection at amino acid level that affects synonymous codon choice [124], [125].

More specifically, rare codons concentrate in mRNA regions that code for transmembrane helical structures [116]. Optimization of codon usage means that organisms match codon usage frequencies with anticodons of common tRNAs [126], [127], [128], [129], [130], [131], [132], [133], speeding translation, affecting cotranslational protein folding [134]. Lopez and Pazos [135] suggest that proper folding into transmembrane structures requires specific spatial kinetics and particular accuracy in the process. Cotranslational protein folding is most apparent on alpha helices and betasheet secondary structures [136], [137], [138], [139], [140]. Hence one expects associations between CDA and these conformational indices of amino acids. Chemical kinetics of the transfer of the amino acid loaded on the tRNA's acceptor stem to the elongating peptide (kinetic estimates from [141]) also constrain codon-anticodon interactions [43].

Following these rationales, CDA might reflect (a) indirectly tRNA synthetase classes and their effects on amino acid positioning during peptide elongation; and (b) directly the spatial kinetics of codon-anticodon interactions, such as tRNA-mRNA approach angles during codon-anticodon duplex formation in the ribosomal translational core(s). These two components should affect according to the cotranslational protein folding hypothesis folding patterns of elongating peptides. Hence CDA is predicted to correlate with amino acid secondary structure conformational parameters for alpha helices, beta turns and/or betasheets (conformational indices are from [142], [143], [144], [145]). The main candidates are the conformational parameters associated with transmembrane foldings (beta turns, and/or parallel and antiparallel betasheets, from references [146], [147]).

## 6 Antiparallel Betasheet Formation and Codon Directional Asymmetry

The hypothesis that CDA associates with cotranslational protein folding predicts correlations between CDA and secondary structure conformation parameters. Betasheets are the major secondary structures found in transmembrane proteins, antiparallel betasheets are more frequent than parallel betasheets [147]. Biases in tRNA synthetase amino acid contents correlate with the amino acid's antiparallel betasheet conformation parameter [148]. Hence, we predict correlations between CDA and conformation parameters, and in particular antiparallel betasheet conformation parameters.

**Fig. 2:**
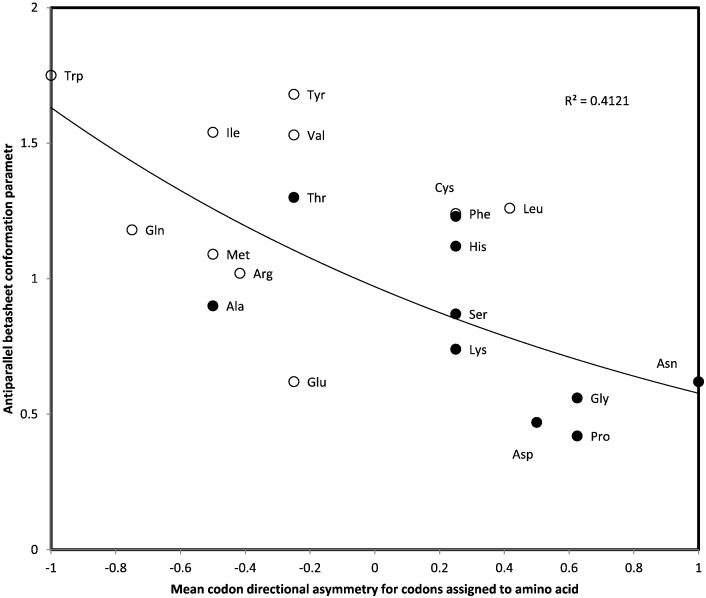
Antiparallel betasheet conformation parameter of amino acids as a function of the mean codon directional asymmetry (CDA) of codons assigned to that amino acid, for the standard genetic code. Amino acids aminoacylated by tRNA synthetases from class I have open circles, filled circles are for tRNA synthetases from class II.

Indeed, antiparallel betasheet conformation parameters correlate negatively with mean CDA of codons assigned to the amino acid according to the standard genetic code (Pearson correlation coefficient r = − 0.642, two-tailed *P* = 0.0023; non-parametric Spearman rank correlation coefficient rs = − 0.564, two-tailed *P* = 0.01; Fig. 2). In contrast, and functioning as a negative control, the correlation between mean CDA and parallel betasheet conformation parameters is not statistically significant (r = − 0.28, two-tailed *P* = 0.23, not shown). The presumed effect of CDA is specific for formation of antiparallel, not parallel, betasheets.

The variation around the regression line is similar for negative and positive CDA ranges (Fig. 2). Hence the determinism of CDA on conformation is comparable for 5′ versus 3′ CDA dominance: effects are independent of coding importance of codon positions. In other words, the ‘information’ in CDA that is relevant to protein secondary structure is similar for asymmetry at first and third codon positions. Alternative scores (Section 4.1) do not change qualitatively the results (*P* values for rs remain above 0.05).

The correlation between mean CDA of codons assigned to amino acids and these amino acids' antiparallel betasheet conformational indices might be due to transitivity, due to associations between CDA and tRNA synthetase classes (see above section) and the association between tRNA synthetase class and conformational indices. In order to control for effects of tRNA synthetase classes, we calculate mean CDA and mean antiparallel betasheet index separately for each tRNA synthetase class. These means are subtracted from CDA and conformational indices of each amino acid in that respective class. These values are residual CDA and conformational indices after excluding effects of tRNA synthetase classes. Residual CDA and residual antiparallel betasheet indices correlate negatively (r = − 0.435, *P* = 0.0275; rs = − 0.461, *P* = 0.0205, one tailed tests). Hence the correlation between CDA and antiparallel betasheet indices is not indirect, through colinearity with tRNA synthetase classes.

The association between CDA and antiparallel betasheet indices has rs with *P* < 0.05 for eight among ten alternative scores (as in Section 4.1) after controlling for tRNA synthetase class. The genetic code seems structured so as to enable synergistic effects of CDA and tRNA synthetase classes on antiparallel betasheet formation, presumably by cotranslational protein folding.

Independently of the correlation between CDA and antiparallel betasheet conformation parameters, a weaker correlation exists between CDA and alpha-helix conformation parameters (r = − 0.556, *P* = 0.011; rs = − 0.499, *P* = 0.05, two-tailed test, not shown). This further correlation confirms that CDA affects protein folding. To our knowledge, these are the first described correlations between a codon property and secondary structure conformational parameters of assigned amino acids. CDA < 0 associates independently with each alpha and antiparallel beta conformational indices, in line with the literature on cotranslational protein folding [136], [137], [138], [139], [140]. Hence according to the working hypothesis, similar kinetic conditions favor each of these two very different secondary structures. Presumably, factors other than CDA (for example chain polarity) determine whether an alpha helix rather than an antiparallel betasheet is initiated during peptide elongation.

## 7 Codon Directional Asymmetry and Prediction of Protein Secondary Structure

The correlation between CDA and conformation parameters might have two causes. First, it could be intrinsic to the genesis of the genetic code, but relatively inconsequent to modern organisms. Secondly, CDA still affects protein folding. In the latter case, correlations between CDA and secondary structure conformation parameters could explain that some synonymous mutations perturb protein function. Indeed, several amino acids have some synonymous codons with opposite CDA, such as for alanine, leucine, serine and valine. Putatively, this would indicate that for these amino acids, synonymous codons with CDA < 0 occur preferentially for mRNA regions coding for antiparallel betasheets, and those with CDA > 0 in other mRNA regions.

Codon usage frequencies are adapted to minimise effects of mutations and translation errors [149], [150], [151], [152]. Hence weighing mean CDA for a given amino acid according to observed synonymous codon usages might increase correlations between CDA and conformation parameters. However, this is not the case for the pool of genes encoded by the human nucleus, nor those coded by the human mitogenome: correlations become in both cases weaker (not shown).

CDAs of stop codons are negative, suggesting a bias for amino acids with high tendencies to participate in antiparallel betasheets when amino acids are inserted at stop codons. Indeed, the evolution of mitochondrial genetic codes seems best reconstructed when assuming insertion of amino acids at stops [153], in line with coevolution between predicted suppressor tRNAs [154], [155], [156] and protein alignment analyses [16], [78], [79], [157], [158], [159], [160], [161]. However, frequencies of amino acids inserted at stops [71], [162], [163], [164], [165], [166] do not significantly correlate with antiparallel betasheet conformation parameters.

This does not mean that associations between synonymous codons in modern mRNAs and secondary structures of modern proteins do not exist. However, this suggests that testing these predictions is not as straightforward as it seems. Among others, secondary structure annotations available in GenBank don't indicate whether a betasheet is parallel or antiparallel. Hence these tests will require involvement of more adequately equipped specialised proteomics teams (for example Caudron and Jestin [147]). Until then, the contribution of CDA for improving secondary structure predictions [26], [167], especially such based on optimization of multiple approaches [168], will remain speculative.

## 8 Mitochondrial Genetic Codes Optimise Codon Directional Asymmetry

Many variant genetic codes are from mitochondria [169]. The reduced mitogenomes almost exclusively encode for mitochondrial transmembrane proteins, which include mainly antiparallel betasheets. In contrast, nuclear genomes encode also for large proportions of cytosolic proteins, which include much fewer betasheets. Hence, we predict that the correlation between CDA and antiparallel betasheet conformation parameters is weaker for genetic codes associated with nucleus-encoded proteomes than for mitochondrial genetic codes. The correlation in Fig. 2 (for the standard genetic code) is calculated for the remaining genetic codes listed by Elzanowski and Ostell [169], after recalculating mean amino acid CDA, considering codon-amino acid reassignments. The correlation's strength for each genetic code is estimated by the Pearson correlation coefficient r.

**Fig. 3:**
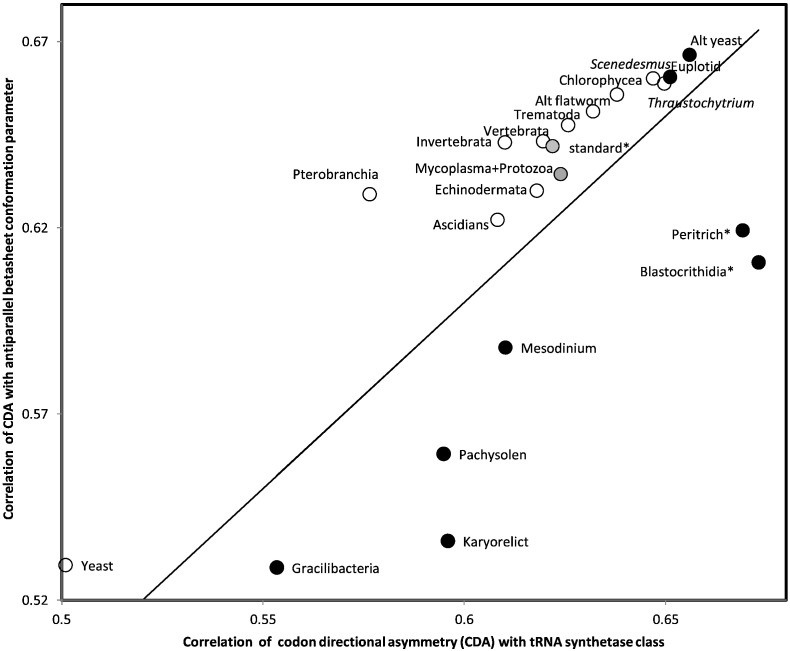
Correlation between antiparallel betasheet conformation parameter of amino acids and mean directional asymmetry (CDA) of codons assigned to that amino acid as a function of the correlation between CDA and the tRNA synthetase class for the corresponding amino acid for different genetic codes. Correlations are Pearson correlation coefficients. Filled/open circles are nuclear/mitochondrial genetic codes, shaded circles are for genetic codes existing in nuclei and mitochondria. The line indicates y = x. Nuclear genetic codes tend to optimise the association between CDA and tRNA synthetase classes, mitochondrial genetic codes tend to optimise the association between CDA and the antiparallel betasheet conformation parameter. Most mitogenome-encoded proteins are transmembrane proteins, hence antiparallel betasheets are particularly frequent in these proteins. Hence genetic code evolution optimises the CDA-antiparallel betasheet association in mitochondria. Open circles: mitochondrial genetic codes; filled circles: nuclear genetic codes; shaded circles: genetic codes used in nuclei and mitochondria.

The correlation between tRNA synthetase classes and CDA is also calculated, by assigning to tRNA synthetase classes I and II values ‘1’ and ‘2’, respectively, and calculating the Pearson correlation coefficients r between this dummy variable representing tRNA synthetase classes and the mean CDA of codons assigned to the corresponding amino acid, for each variant genetic code. The CDA-antiparallel betasheet correlation coefficients are plotted as a function of the CDA-tRNA synthetase class correlation coefficients for the various genetic codes (Fig. 3). The line in Fig. 3 indicates y = x, meaning that both correlations have equal strengths. Note that in context of this particular section, Pearson correlation coefficients are used as quantitative estimates of the strength of a correlation, not as test statistics to infer that a correlation exists.

All eleven mitochondrial genetic codes have stronger correlations between CDA and antiparallel betasheet conformation parameters than between CDA and tRNA synthetase classes. Obtaining this result for all eleven mitochondrial genetic codes has *P* = 0.00049 (two-tailed sign test). Two additional genetic codes occur in nuclear and mitochondrial genomes: the standard genetic code, and the *Mycoplasma*/*Spiroplasma* genetic code that also occurs in mold, protozoan and coelenterate mitochondria. These two genetic codes follow the pattern observed for the eleven genetic codes only found in mitochondria.

Six among eight genetic codes associated only with nuclear genomes are below the line y = x in Fig. 2, indicating that the CDA-tRNA synthetase class correlation is frequently a greater constraint for nuclear genetic codes than mitochondrial ones. This qualitative difference between nuclear and mitochondrial genetic codes has *P* = 0.001 (two-tailed Fisher exact test). This divide might reflect different constraints on protein folding for populations of mitochondrion-encoded versus nucleus-encoded proteins. This pattern might indicate stronger synergy between effects of CDA and tRNA synthetase class on cotranslational folding for nucleus-encoded proteins translated in the cytosol than mitogenome-encoded ones.

These results indicate that associations between CDA and conformation parameters, and between CDA and tRNA synthetase classes, drive differentially evolutions of mitochondrial versus nuclear genetic codes. Tentatively, amino acid positioning on the tRNA acceptor stem is less relevant for mitochondrial translation than CDA, the opposite is true for cytosolic translations.

## 9 Whole Organism Properties and Codon Directional Asymmetry

Whole organism properties correlate sometimes with molecular properties [170], [171]: morphological versus molecular rates of evolution [172], [173], [174]; growth rates and genome sizes [175], [176], [177], [178]; and metabolic costs of protein synthesis [179]; body temperatures and predicted expanded codons [180], [181], [182], [183]; developmental stability estimated by lateral differences between bilateral morphological traits and accuracy of various aspects of molecular processes, such as replication [184], ribosomal translation [20], [185], and tRNA loading [14]. CDA might also correlate with whole organism properties.

### 9.1 Lepidosaurian Body Temperature and Codon Directional Asymmetry

**Fig. 4:**
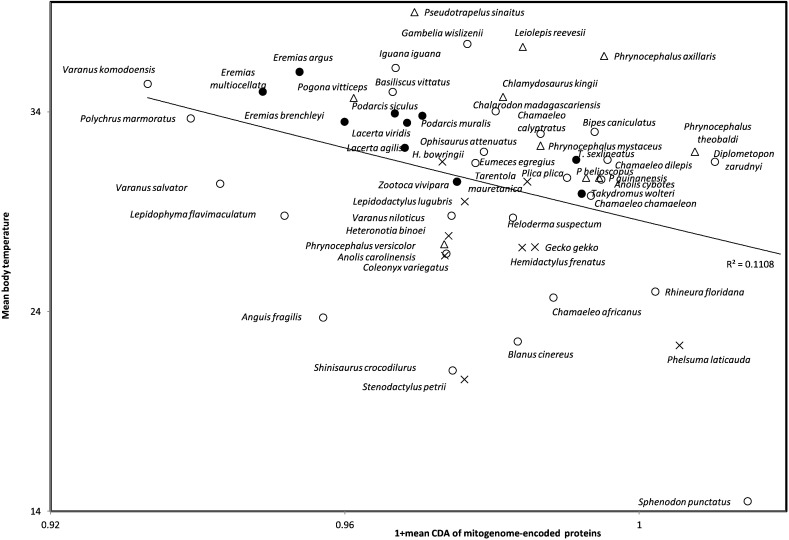
Lepidosaurian body temperature as a function of mean codon directional asymmetry of codons in protein coding genes encoded by complete mitogenomes available in GenBank. Compilation of body temperatures and mitochondrial genomes as in Table 1 of Seligmann and Labra [183]. Agamidae are indicated by triangles, Gekkota by crosses and Lacertidae by filled circles. Species from various other families have open circles.

Temperature reflects noise in molecular movements, potentially affecting contranslational protein folding, which indeed depends on optimal temperatures [186]. Hence, formation of antiparallel betasheets might be impeded by high temperatures. Therefore, we expect that negative CDAs promote betasheet formation despite high temperature. Hence when comparing the mean CDA calculated across all 13 membrane-embedded mitogenome-encoded proteins of different organisms, we expect that organisms with high temperatures have low mean CDA for the same homologous genes. Indeed, the mean CDA of lepidosaurian mitochondrion-encoded proteins decreases with their body temperature (ro = − 0.283, one tailed *P* = 0.018, Fig. 4, temperature data compiled for species with complete mitogenome available in GenBank by Seligmann and Labra [183], therein Table 1).

This correlation is also statistically significant within the family Lacertidae (ro = − 0.842, one-tailed *P* = 0.001). It is negative for Agamidae (ro = − 0.255, one-tailed *P* = 0.238), Gekkota (ro = − 0.25, one-tailed *P* = 0.258), iguanid lizards (ro = − 0.333, one-tailed *P* = 0.21), Varanidae (ro = − 1.00, one-tailed *P* = 0.005) and *Chamaeleo* (ro = − 0.40, one-tailed *P* = 0.30) and for the pool of remaining isolated species from various families (*Heloderma*, *Shinisaurus*, *Lepidophyma*, *Sphenodon* (ro = − 0.238, one tailed *P* = 0.285). The correlation is positive for Amphisbaenia (ro = 0.40, one tailed *P* = 0.30). Hence seven among eight phylogenetically independent samples yield negative correlations, which is a significant majority according to a sign test (*P* = 0.0176). Considering the qualitative direction of correlations for phylogenetically independent species samples follows the principle of phylogenetically independent contrasts [187]. This confirms that positive results are not confounded by phylogenetic inertia among species. Results of this sign test are valid independently of *P* value adjustments for multiple tests.

GC contents could confound this correlation, because G:C base pairs are linked by three hydrogen interactions, while A:T and A:U base pairs by only two hydrogen bridges. Hence GC contents usually increases with temperature, as it confers higher stability to structures formed by nucleotide chains [188], [189], [190]. However, GC codon content does not correlate with body temperature for mitochondria of the above mentioned lepidosaurian species (r = − 0.0425, one-tailed *P* = 0.379). This is in line with results from various analyses [191], [192], [193]; that didn't detect the expected GC-temperature correlation. This negative control stresses that the association in Fig. 4 is not trivial.

### 9.2 Developmental Stability and CDA

Molecular noise (in terms of erratic molecular movements) affecting mitochondrial transmembrane protein folding might cause developmental inaccuracies at the whole organism level. Hence, we explore the correlation between mean CDA of mitogenome-encoded proteins and developmental stability of the 4th toe of Lepidosauria, estimated by the Pearson correlation coefficient r between subdigital lamellae counts on left and right sides (data from [194], [195], [196], [197], [198]). Developmental stability/accuracy decreases with mean CDA of mitogenome-encoded proteins (ro = − 0.316, one-tailed *P* = 0.0235), as expected by the working hypothesis. However, analyzing separately species grouped according to phylogenetic groups (as in previous section) yields negative correlations only in five among eight groups, which is not statistically significant at *P* < 0.05 according to a one sided sign test. Hence this preliminary result on CDA and developmental stability is at best tentative.

### 9.3 Lifespan and CDA

**Fig. 5:**
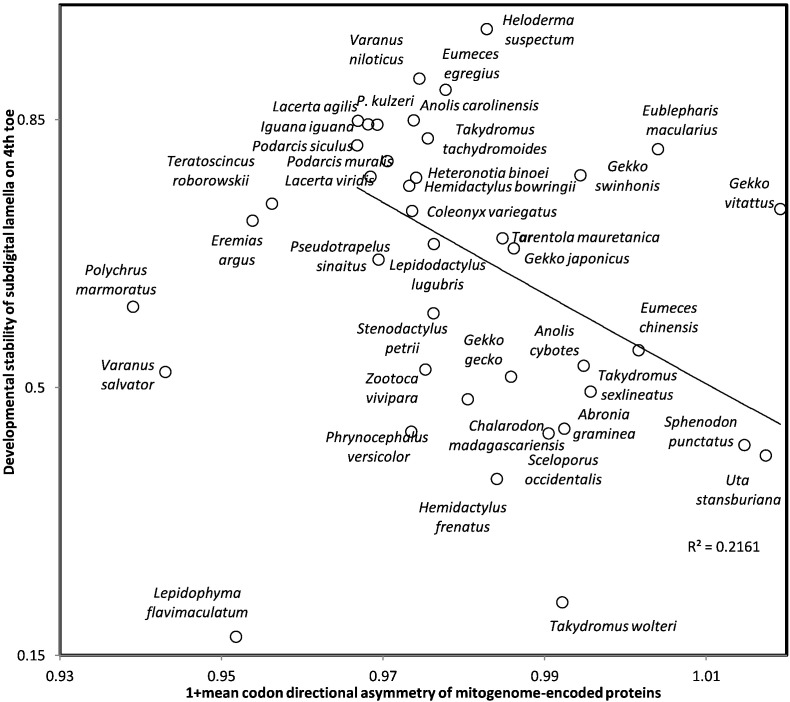
Developmental stability of bilateral counts of subdigital lamellae on 4th toe of Lepidosauria (estimated by Pearson correlation coefficients r between counts on left and right sides) as a function of mean codon directional asymmetry (CDA) of codons in all 13 genes of mitogenome-encoded transmembrane proteins.

Patterns between CDA and temperature, and CDA and developmental stability (Fig. 4, Fig. 5) suggest that CDA < 0 for mitogenome-encoded proteins associates with longevity. For this purpose, we compared codon contents in mitogenomes of 112 semi-supercentenarians and 96 centenarians versus those of 97 healthy young controls [199], [200] (Table 3). Codons with CDA = − 1 are more frequent in supercentenarians than in controls for seven among eight comparisons, which is a significant majority according to a one tailed sign test (*P* = 0.0176). No tendencies are observed for other CDA values (− 0.5, 0, 0.5, 1), nor for comparisons between centenarians and controls. The result is suggestive that CDA < 0 could contribute to extreme longevity, but the high number of tests and the small differences in codon frequencies stress cautious interpretation.

Overall, analyses weakly confirm predictions for correlations between CDA and whole organism properties (body temperature, developmental stability, longevity). These suggest that analyses considering additional information, such as residue-specific location in three dimensional protein structures, might yield positive results. More up-to-date methods for including phylogenetic information in relation to evolutionary adaptive optima might also alter conclusions [201].

### 9.4 Replicational Deamination Gradients

Mitochondrial DNA replication differs from nuclear chromosome replication [202] and is usually strand asymmetric resulting in replicational deamination gradients where C → T and A → G substitutions exceed reversed mutations proportionally to the time spent single stranded during replication [203], [204], [205], [206], [207], [208]. Inverting the direction of the light strand replication origin also inverts the direction of the replicational deamination gradients [209], [210], [211], [212], [213], [214], [215], [216], [217]). These physico-chemical mutation pressures could affect CDA according to gene locations on the mitogenome, independently of protein properties.

Mean CDA of the 13 human mitogenome-encoded proteins does not correlate with time spent single stranded by that gene during replication, assuming light strand replication initiates at the OL, the light strand replication origin (ro = 0.033, *P* = 0.92, two tailed test). DNA templating for tRNA genes presumably also functions sometimes as replication origins [184], [218], [219]. Integrating the possibility of these multiple replication origins yields gene-wise single-strand durations that converge with transcriptional singlestrandedness [220]. The correlation between transcriptional duration of singlestrandedness and mean gene CDA is also not statistically significant (ro = 0.418, *P* = 0.156, two tailed test). Hence, we do not detect statistically significant effects of mutation pressures on mean CDA of human mitochondrial genes.

### 9.5 Adjusting Statistical Significances for Multiple Tests

Analyses that include several tests have to adjust *P* values according to the number of tests. This is because, when deciding that a result is positive at *P* < 0.05, when k tests are performed, on average, k × 0.05 tests are false positives. Bonferroni's correction considers that when performing k tests, results are statistically significant at *P* = 0.05 for any specific test among k tests if *P* < 0.05/k. This correction is reputedly overconservative [221], [222]. Unadjusted *P*s minimise risks of false negative results, Bonferroni's method minimises risks of false positives. The Benjamini-Hochberg adjustment for false discovery rates [223] optimises between these two risks and seems most adequate [224]. This method ranks all k *P* values from highest to lowest (best), adjusted *P*s are the product of *P* with k divided by the rank i, where i ranges from 1 to k. This means that the ‘best’ (lowest) *P* is unchanged, and that the ‘worst’ (highest) *P* value after adjustment follows Bonferroni's adjustment. *P*s with intermediate rank are intermediate between these extremes.

Here we consider only *P* values from non-parametric tests, when also parametric tests were done. For some of the associations described, more than one test was done, but these are then summarised by a test that integrates the previous tests. Adjustments consider in these cases only the latter *P* value. Along this approach a total of 29 hypothesis tests were done, as detailed in Table 2. Control analyses (such as with GC contents, and mutational gradients, in total 29 tests) are also included in the list of multiple tests. These are not related to the main CDA hypothesis and could arguably be excluded. Excluding controls does not alter qualitatively results of the adjustments of *P* values.

**Table 2:** Benjamini-Hochberg adjustment of *P* values of non-redundant hypothesis tests. The total number of tests including controls is 58 (Rank 1 and adjusted P1), excluding controls and considering only tests pertaining directly to CDA, there are 29 tests (rank 2 and adjusted P2). Only tests with unadjusted *P* < 0.05 are shown, all these tests pertain to CDA directly.

Test	P	Rank 1	Adj P1	Rank 2	Adj P2
Number of mitochondrial genetic codes above line in Fig. 3	0.00050	58	0.00050	29	0.00050
Number of mitochondrial genetic codes above line vs number of nuclear codes below line in Fig. 3	0.00100	57	0.00102	28	0.00100
tRNA synthetase classes and CDA	0.00200	56	0.00207	27	0.00207
tRNA synthetase classes and mean CDA of codons assigned to amino acids	0.00295	55	0.00312	26	0.00319
Contacts between Fujimoto's tetrahedron faces	0.00960	54	0.01031	25	0.01075
Correlation CDA-antiparallel betasheet indices	0.01000	53	0.01094	24	0.01167
Absolute CDA and circular code	0.01600	52	0.01785	23	0.01948
Temperature and mean CDA of 13 lepidosaurian mitogenome-encoded proteins	0.01760	51	0.02002	22	0.02240
Human lifespan and mitochondrial codon usages-CDA	0.01760	50	0.02042	21	0.02347
Contacts within Fujimoto's tetrahedron faces	0.01800	49	0.02131	20	0.02520
Partial correlation CDA-antiparallel betasheet indices	0.02050	48	0.02477	19	0.03021
CDA-developmental stability	0.02350	47	0.02900	18	0.03656
Absolute CDA-reading frame retrieval capacity	0.02600	46	0.03278	17	0.04435
Alpha helix-CDA	0.05000	45	0.06444	16	0.08750

**Table 3:** Mean codon frequencies (promil) in the 13 mitogenome-encoded genes of three groups of Japanese males: 97 healthy controls, 112 semi-supercentenarians and 96 centenarians from references [199], [200].

Codon	CDA	Control	Super	Cent	Codon	CDA	Control	Super	Cent
UUU	0	20.34	20.32	20.34	UAU	0.00	12.11	12.13	12.15
UUC	0.5	36.59	36.61	36.60	UAC	− 0.50	23.39	23.35	23.35
UUA	1	19.07	19.04	19.01	UAA	− 1.00	2.09	2.09	2.10
UUG	1	4.59	4.57	4.57	UAG	− 0.50	0.81	0.81	0.80
CUU	− 0.5	17.02	17.03	17.00	CAU	0.50	4.71	4.71	4.71
CUC	0	43.89	43.88	43.89	CAC	0.00	20.80	20.80	20.79
CUA	0.5	72.88	72.87	72.90	CAA	− 1.00	21.61	21.61	21.59
CUG	0.5	11.74	11.78	11.79	CAG	− 0.50	2.08	2.09	2.10
AUU	− 1	33.06	33.03	33.07	AAU	1.00	8.38	8.39	8.41
AUC	− 0.5	51.34	51.30	51.25	AAC	1.00	34.72	34.74	34.72
AUA	0	43.77	43.81	43.82	AAA	0.00	22.37	22.40	22.39
AUG	− 0.5	10.72	10.74	10.73	AAG	0.50	2.63	2.61	2.61
GUU	− 1	8.19	8.19	8.18	GAU	0.50	3.89	3.92	3.93
GUC	− 0.5	12.61	12.64	12.68	GAC	0.50	13.43	13.38	13.38
GUA	0.5	18.39	18.40	18.36	GAA	− 0.50	16.87	16.85	16.86
GUG	0	4.71	4.71	4.73	GAG	0.00	6.23	6.25	6.24
UCU	0	8.42	8.42	8.41	UGU	0.00	1.30	1.30	1.31
UCC	− 0.5	25.99	25.99	26.00	UGC	0.50	4.50	4.50	4.50
UCA	0.5	21.81	21.81	21.80	UGA	− 0.50	24.48	24.46	24.42
UCG	0.5	1.80	1.79	1.80	UGG	− 1.00	2.91	2.92	2.97
CCU	0.5	10.70	10.66	10.68	CGU	− 0.50	1.80	1.80	1.80
CCC	0	31.39	31.43	31.42	CGC	0.00	6.80	6.80	6.80
CCA	1	13.71	13.71	13.72	CGA	− 0.50	7.40	7.40	7.40
CCG	1	1.78	1.78	1.78	CGG	− 1.00	0.50	0.50	0.50
ACU	− 0.5	13.41	13.39	13.43	AGU	0.50	3.70	3.69	3.69
ACC	− 1	40.44	40.45	40.36	AGC	0.50	10.26	10.27	10.29
ACA	0	34.80	34.78	34.77	AGA	0.00	0.30	0.29	0.30
ACG	0.5	2.57	2.58	2.58	AGG	− 0.50	0.30	0.30	0.30
GCU	− 0.5	11.73	11.73	11.74	GGU	1.00	6.30	6.32	6.33
GCC	− 1	32.55	32.56	32.60	GGC	1.00	22.88	22.87	22.85
GCA	− 0.5	21.60	21.58	21.60	GGA	0.50	18.00	18.02	18.03
GCG	0	2.08	2.09	2.08	GGG	0.00	8.55	8.52	8.51

The analysis for codon usage associated with lifespan includes 10 tests (for CDA values − 1, − 0.5, 0, 0.5, and 1, and this for comparisons between controls and centenarians, and between controls and supercentenarians). Among unadjusted *P* values with *P* < 0.05, only the adjusted *P* value for the correlation between mean CDA of codons assigned to amino acids and the amino acids' alpha helix conformational indices is above 0.05. This occurs when considering all 58 tests, and when considering only the 29 tests directly pertaining to the working hypothesis about CDA. Qualitatively, results of *P* adjustments are robust in relation to numbers of tests included in this analysis: for example, for *P* with rank 17 to get *P* > 0.05 after adjustment, one requires k = 89 when including negative controls and k = 33 when excluding negative controls. Hence even if one was to increase numbers of tests included in the analyses, the relevant cutoff property of the distribution of adjusted *P*s is relatively robust, so that issues related to multiple tests are unlikely to alter conclusions.

## 10 A New Directional Codon Dimension

Intuitively, it seems conceivable that CDA, via its plausible effects on codon-anticodon interactions, affects cotranslational protein folding. However, developing a mechanistic scenario that explains why this effect should occur for antiparallel betasheets rather than parallel ones, or for alpha helices, is more difficult. We propose that some (unspecified) conformations depend on translational speed. Other conformations might be favored by random movements of the tRNA's loaded acceptor stem in relation to the elongating peptide, versus more directed movements of that stem, hence some ratio between kinetic noise and direction. Our educated guess (but nothing beyond that) is that CDA relates more to the latter type of mechanisms. We also lack clues on why CDA < 0 promotes antiparallel betasheets, and CDA > 0 prevents them. Alpha helices might be more simple structures that require less order than antiparallel betasheets. A similar rationale might function for parallel and antiparallel betasheets. In addition, the ratio between parallel and antiparallel betasheets is about 1:7 [26]: the genetic code might be optimised towards ‘coding’ for the most frequent protein conformation.

The genetic code can be characterised as a hypercomplex mathematical multidimensional symmetry structure [225]. In other terms, the genetic code reminds spontaneously self-organizing structures such as crystals [226], [227]. Crystals result from specific rules organizing relations between atoms. Similarly, but at a much higher level of molecular complexity, the genetic code organises relations between nucleic and amino acid sequences. The genetic code might be thought as an imaginary polyhedron with 64 triangular faces (64 codons with three nucleotide positions). The geometrical form of this structure remains unknown, but several symmetries implied by RNA/DNA structure and chemistry are known, such as reverse-complementarity (implied by the double helix structure), and the purine-pyrimidine as well as the alpha-keto groupings of nucleic acids. Formulation of a generalised description of this complex structure is a difficult task. It is simplified by projections of the complex structure on specific scales/planes of probable biological interest.

Here learned intuition detects a new symmetry property, based on codon content directionality. Analyses here can be seen as projecting that complex genetic code structure on the CDA scale, enabling to detect some new properties of the genetic code. The details of the scale of CDA scores as presented here is probably inaccurate and will hopefully be amended. CDA implies that a directional dimension that had not been apprehended links codons and amino acids: biologically meaningful information relating to protein structure is embedded in the comparison between codons and their reversed (not reverse-complemented) sequence. This palindrome-minded approach to codons probably reflects error-correcting properties of primitive genetic code(s) [228].

## 11 Conclusions

A property of codons, codon directional asymmetry (CDA), is defined for the genetic code. Codons are classified into symmetric (CDA = 0), 5′- and 3′-asymmetric (negative and positive CDA). CDA maps non-randomly on Fujimoto's tetrahedral representation of the genetic code. Symmetric codons are the most common codons in frame-error-correcting codes, such as comma-free and circular codes. Most codons assigned to amino acids aminoacylated to cognate tRNAs by tRNA synthetases class I have CDA < 0, those assigned to cognates of tRNA synthetases class II have usually CDA > 0.

Amino acid tendencies to participate in antiparallel betasheets decrease with CDA. Results suggest that CDA and tRNA synthetase class affect spatial kinetics of peptide elongation. These spatial kinetics affect local peptide elongation rates, which determine cotranslational peptide folding during peptide synthesis. Hence CDA, a property of gene sequences, bears useful information to predict protein folding. Some synonymous codons have CDA with opposite signs, potentially explaining how some synonymous mutations alter protein function.

CDA probably played a role in the evolution of genetic codes. Mitochondrial genetic codes optimise associations between CDA and antiparallel betasheet formation, nuclear genetic codes tend to optimise associations between CDA and tRNA synthetase class. This difference might mean that synergistic effects of CDA and tRNA synthetase class on cotranslational protein folding are stronger for nuclear than mitochondrial genetic codes. CDA affects codon-amino acid (re)assignments, hence plays an important role in genetic code evolution.

Preliminary analyses suggest that average CDA of mitochondrion-encoded proteins decreases with body temperature, increases developmental stability and lifespan, but further controlled analyses are required to confirm these potential whole organism effects of codon directional asymmetry (CDA).

## Conflicts of Interests

None.
